# Metformin Treatment Does Not Inhibit Growth of Pancreatic Cancer Patient-Derived Xenografts

**DOI:** 10.1371/journal.pone.0147113

**Published:** 2016-01-13

**Authors:** Matthew B. Lipner, Raoud Marayati, Yangmei Deng, Xianxi Wang, Laura Raftery, Bert H. O’Neil, Jen Jen Yeh

**Affiliations:** 1 Department of Pharmacology, School of Medicine, The University of North Carolina, Chapel Hill, NC, United States of America; 2 Lineberger Comprehensive Cancer Center, The University of North Carolina, Chapel Hill, NC, United States of America; 3 ProHealth Care Regional Cancer Center, Waukesha, WI, United States of America; 4 Department of Medicine, Division of Medical Oncology, University of Indiana, Indianapolis, IN, United States of America; 5 Department of Surgery, Division of Surgical Oncology and Endocrinology, The University of North Carolina, Chapel Hill, NC, United States of America; Université de Sherbrooke, CANADA

## Abstract

There is currently tremendous interest in developing anti-cancer therapeutics targeting cell signaling pathways important for both cancer cell metabolism and growth. Several epidemiological studies have shown that diabetic patients taking metformin have a decreased incidence of pancreatic cancer. This has prompted efforts to evaluate metformin, a drug with negligible toxicity, as a therapeutic modality in pancreatic cancer. Preclinical studies in cell line xenografts and one study in patient-derived xenograft (PDX) models were promising, while recently published clinical trials showed no benefit to adding metformin to combination therapy regimens for locally advanced and metastatic pancreatic cancer. PDX models in which patient tumors are directly engrafted into immunocompromised mice have been shown to be excellent preclinical models for biomarker discovery and therapeutic development. We evaluated the response of four PDX tumor lines to metformin treatment and found that all four of our PDX lines were resistant to metformin. We found that the mechanisms of resistance may occur through lack of sustained activation of adenosine monophosphate-activated protein kinase (AMPK) or downstream reactivation of the mammalian target of rapamycin (mTOR). Moreover, combined treatment with metformin and mTOR inhibitors failed to improve responses in cell lines, which further indicates that metformin alone or in combination with mTOR inhibitors will be ineffective in patients, and that resistance to metformin may occur through multiple pathways. Further studies are required to better understand these mechanisms of resistance and inform potential combination therapies with metformin and existing or novel therapeutics.

## Introduction

Pancreatic cancer is one of the most aggressive and lethal malignancies, with 80% of patients presenting with locally advanced or metastatic disease that portends a 6–12 month median survival and a dismal 6% five-year survival rate [1]. Chemotherapy produces only modest improvements in survival, and novel therapies are desperately needed to improve treatment options for this large patient population [2]. There is currently tremendous interest in developing anti-cancer therapeutics that target cell signaling pathways important in both cell metabolism and cell growth [3]. The 5' adenosine monophosphate-activated protein kinase (AMPK) pathway has gained increasing interest, as AMPK physiologically inhibits the mammalian target of rapamycin (mTOR) to maintain homeostasis in conditions of decreased available cellular energy sources [4, 5]. Studies have shown that mTOR signaling plays key roles in survival and proliferation of malignant cells [6, 7]. Thus, AMPK activators have generated substantial interest as potential antineoplastic agents that function by altering metabolism and inhibiting the mTOR pathway [3].

Metformin is the first-line agent for treatment of type 2 diabetes mellitus. Metformin inhibits mitochondrial oxidative phosphorylation, thereby increasing the ratio of AMP to ATP [8, 9]. High levels of AMP activate AMPK, which then inhibits energy-consuming pathways such as protein synthesis, in part by downregulating mTOR signaling by direct phosphorylation of the tumor suppressor TSC2 and the mTOR binding partner Raptor [9–13]. The state of energy conservation induced by metformin has been proposed to explain the cytostatic effect of metformin on cancer [9] and the apparent protective effect observed in diabetic patients treated with metformin who subsequently develop pancreatic cancer [14].

Several epidemiological studies have indicated that patients with diabetes taking metformin have a decreased incidence of pancreatic cancer [14–17]. This has prompted a great deal of excitement to evaluate metformin, a widely used drug with negligible toxicity, as a therapeutic modality in pancreatic cancer. There are currently 3 clinical trials evaluating metformin in combination with various chemotherapies in pancreatic cancer (cancer.gov/clinicaltrials). Preclinical studies in cell line xenografts and one recent study in patient-derived xenograft (PDX) models have shown promise [18–22].

PDX models in which patient tumors are directly engrafted into immunocompromised mice have been shown to recapitulate primary tumor architecture and genetic characteristics, even after passaging and expanding the tumors in successive generations of mice [23, 24]. Furthermore, PDX models are superior to traditional cell line xenografts, which are adapted to in vitro growth and lack the heterogeneity of patient tumors, for evaluating responses to therapies and novel biomarkers [23–27]. Until recently, there have been very limited studies of PDX responses to many proposed oncological agents, and results for metabolic therapies like metformin are still severely lacking [27]. Thus, the objective of this study was to evaluate the response of pancreatic cancer PDX models to metformin and to investigate metformin’s mechanism of action and compensatory resistance pathways.

## Materials and Methods

### Drugs and reagents

Metformin hydrochloride (Spectrum, New Brunswick, NJ, USA) was dissolved in phosphate-buffered saline (PBS) for both in vitro and in vivo studies. Rapamycin (LC Laboratories, Woburn, MA, USA) and BEZ235 (Center for Integrative Chemical Biology and Drug Discovery, UNC Eshelman School of Pharmacy, Chapel Hill, NC, USA) were dissolved in dimethyl sulfoxide (DMSO) for in vitro combination therapy studies. Antibodies against phosphorylated AMPKα (Thr172), AMPKα, AMPKα1, AMPKα2, phosphorylated mTOR (Ser2448), mTOR, phosphorylated p70S6K (Thr389), p70S6K, phosphorylated 4E-BP1 (Thr37/46), and 4E-BP1 were from Cell Signaling (Beverly, MA, USA). Anti-glyceraldehyde phosphate dehydrogenase (GAPDH) and horseradish peroxidase-conjugated goat anti-rabbit IgG were from Santa Cruz Biotechnology (Santa Cruz, CA, USA). Pierce® ECL Western Blotting Substrate was from Thermo Scientific (Rockford, IL, USA). Apo-ONE Homogeneous Caspase-3/7 assay kit was from Promega (Madison, WI, USA).

### Cell culture and transduction with lentivirus

Pancreatic cancer cell lines Capan-2, CFPAC-1, HPAF-II, and SW1990 were obtained from the American Type Culture Collection (ATCC), authenticated via short–tandem repeat (STR) profiling (Genetica, Burlington, NC, USA), and tested negative for mycoplasma by indirect staining. Cell lines were cultured in RPMI 1640 medium supplemented with 10% fetal bovine serum (FBS), 100 U/ml penicillin, and 100 μg/ml streptomycin (Invitrogen, Carlsbad, California, USA) at 37°C in a humidified 5% CO2 atmosphere.

The puromycin domain of the AMPKα1–859 pLKO.1 reporter plasmid (generously donated by the laboratory of Channing Der, PhD at The University of North Carolina, Chapel Hill, NC, USA) was replaced with a blasticidin domain by restriction enzyme digestion with BamHI and KpmI. The second generation replication-incompetent lentivirus was generated in 293T cells with a four-plasmid system: the reporter plasmid, pMDL gag/pol RRE, pRSV-Rev, and pCMV VSV-G. For transduction with lentivirus, 1×10^6^ CFPAC-1 and HPAF-II cells were seeded in 100 mm plates with lentivirus and a final polybrene concentration of 8 μg/mL. After 24 hours, the medium was replaced, and the cells were cultured for another 4 days with 2 μg/ml of puromycin or 10 days with 10 μg/ml of blasticidin. The cells were trypsinized and analyzed by western blotting to determine gene knockdown.

The expression vector for myc-mTOR transient overexpression was obtained from Addgene (plasmid 1861, Cambridge, MA, USA). Transfection of 5x10^5^ CFPAC-1 or HPAF-II cells was carried out with Lipofectamine 2000 (Invitrogen, Carlsbad, California, USA) using manufacturer guidelines. Following 24 hour incubation, transfected cells were treated with 5 mM metformin for an additional 24 hours, at which point cells were washed with PBS, harvested by scraping, and stored at −80°C until protein isolation.

### MTT assay for cell proliferation

To determine cell viability following drug treatments, 5x10^3^ cells were plated in quadruplicate into 96-well plates in 200 μl of RPMI 1640 medium and cultured overnight. The medium was then replaced with fresh medium containing either PBS as a vehicle control, metformin (0–5 mM), or metformin plus either rapamycin (0–80 μM) or BEZ235 (0–1 μM). After an additional 48 hour incubation, 50 μl of 5 mg/mL 3-(4,5-dimethylthiazol-2-yl)-2,5-diphenyl tetrazolium bromide (MTT) dissolved in PBS at pH 7.4 was added to each well. Following 1 hour incubation, the culture medium and MTT reagent was aspirated and 200 μl of dimethyl sulfoxide was added to each well and mixed thoroughly. The absorbance at OD560 nm was measured using a Synergy 2 plate reader (BioTek, Winooski, VT, USA). Relative proliferation at each drug concentration was calculated according to the formula: 100% * (experimental OD560 / vehicle OD560). Statistical significance was determined using one-way ANOVA analysis with Dunnett’s multiple comparisons test. For combination therapy studies, synergy was assessed using Compusyn software employing the Chou-Talalay median effect principle (ComboSyn, Inc. Paramus, NJ, USA). All assays were performed in triplicate.

### Western blot conditions

After the indicated time of incubation with metformin, cells were washed with PBS, harvested by scraping, and then lysed in 200 μL RIPA buffer containing 50 mM Tris-HCl (pH 7.4), 150 mM NaCl, 1 mM EDTA, 1% Triton X, 1 mM NaF, and 0.25% Na deoxycholate and protease inhibitors. Protein extracts (30 μg) were electrophoresed on 10% SDS polyacrylamide gels and electrotransferred to polyvinylidene difluoride (PVDF) membranes. For determination of knockdown of AMPKα1 and AMPKα2, membranes were blocked with 5% non-fat dried milk in Tris-buffered saline and then incubated overnight at 4˚C with 1:1000 dilutions of anti-AMPKα1, anti-AMPKα2, and anti-AMPKα antibodies. For cell and tissue lysates isolated following metformin treatments, membranes were incubated at 4°C overnight with 1:1000 dilutions of anti-phospho-mTOR, anti-mTOR, anti-phospho-p70S6K, anti-p70S6K, anti-phospho-4E-BP1, anti-4E-BP1, anti-phospho-AMPKα, and anti-AMPK antibodies. Membranes were then washed and incubated with a 1:5000 dilution of horseradish peroxidase-conjugated goat anti-rabbit secondary antibody (Santa Cruz Biotechnology, Santa Cruz, CA, USA). Immunoreactive bands were detected by chemiluminescence using the Pierce® ECL Western Blotting Substrate. Intensity of each immunoreactive band was quantified by densitometry using Image J software (NIH, Bethesda, Maryland, USA), and expressed relative to the PBS-treated cells or mice. GAPDH was used to ensure equivalent protein loading. Statistical significance was determined using Student’s *t*-tests for two sample comparisons and one-way ANOVA analysis with Dunnett’s multiple comparisons test for three or more sample comparisons.

### PDX cohort expansion

Pancreatic ductal adenocarcinoma tissue from de-identified patients with localized pancreatic cancer who underwent curative surgical resection were obtained from the University of North Carolina Institutional Review Board (IRB) approved Tissue Procurement Facility after IRB approval (08–1153). Tumor tissue was engrafted subcutaneously into the flanks of NSG/NOD mice, expanded, and passaged over time, as described previously [28, 29]. 7–8 week old Nu/nu mice with an average weight of 18–20 g were used in all experiments. All animal experiments were carried out in accordance with the U.S. National Institutes of Health (NIH) Guide for the Care and Use of Laboratory Animals under protocols approved by the University of North Carolina Institutional Animal Care and Use Committee (12–314).

### Metformin treatment of PDX cohort

Treatment was started after engrafted tumors grew to a median tumor size of 108 mm^3^. At least five mice were included in each treatment group for each PDX tumor line. Mice were treated with metformin (200 or 400 mg/kg) or PBS (20 μl/g) by once daily oral gavage. The first day of treatment was designated as day 0 and treatment was continued for 28 days. Tumor volume (V) was measured twice per week using calipers, and calculated as (length×width^2^)/2. Animal body weights were measured once weekly. Statistical significance was determined using one-way ANOVA analysis with Dunnett’s multiple comparisons test. Tumor tissue was collected two hours after the last treatment and cut into two parts: one part was snap-frozen in liquid nitrogen and stored at −80°C until protein isolation, while the second part was fixed in 10% formalin and paraffin embedded (FFPE). FFPE tissue blocks were sectioned and stained with hematoxylin and eosin for histopathological evaluation.

## Results

### Metformin does not inhibit growth of PDX tumors

**Fig 1:**
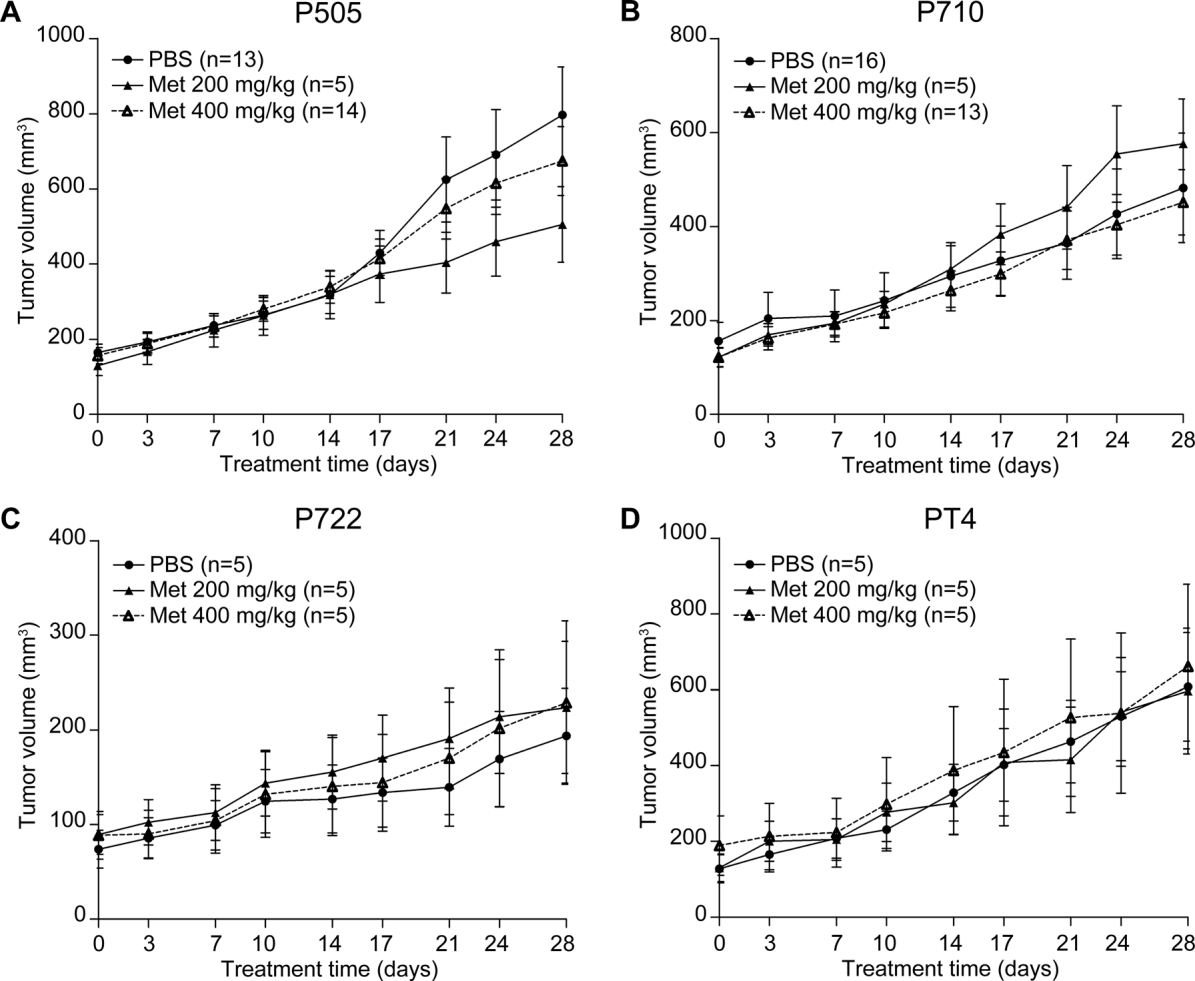
Metformin does not inhibit growth of PDX tumors. No significant growth inhibition was observed in four different pancreatic cancer PDX tumor lines at any time point during a 28 day treatment course with 200 mg/kg or 400 mg/kg metformin administered by daily oral gavage.

We evaluated the response of four pancreatic cancer PDX tumor lines to metformin (200 and 400 mg/kg) for 28 days. These doses were chosen as higher doses have been shown to be necessary in mice to produce a decrease in blood glucose in diabetic animals [19, 30, 31]. In addition, higher doses of metformin (0.1% w/w) have been shown to increase the longevity of mice [32]. No tumor growth inhibition or regression was seen in any of the four PDX tumor lines at any time point measured (Fig 1). No change in body weights occurred over the course of the study and tumor architecture remained grossly unchanged following 28 days of treatment as assessed by hematoxylin and eosin staining (S1 Fig).

### Activation of AMPK and inhibition of p70S6K phosphorylation in PDX tumors is not sustained after a 28 day treatment with metformin

**Fig 2:**
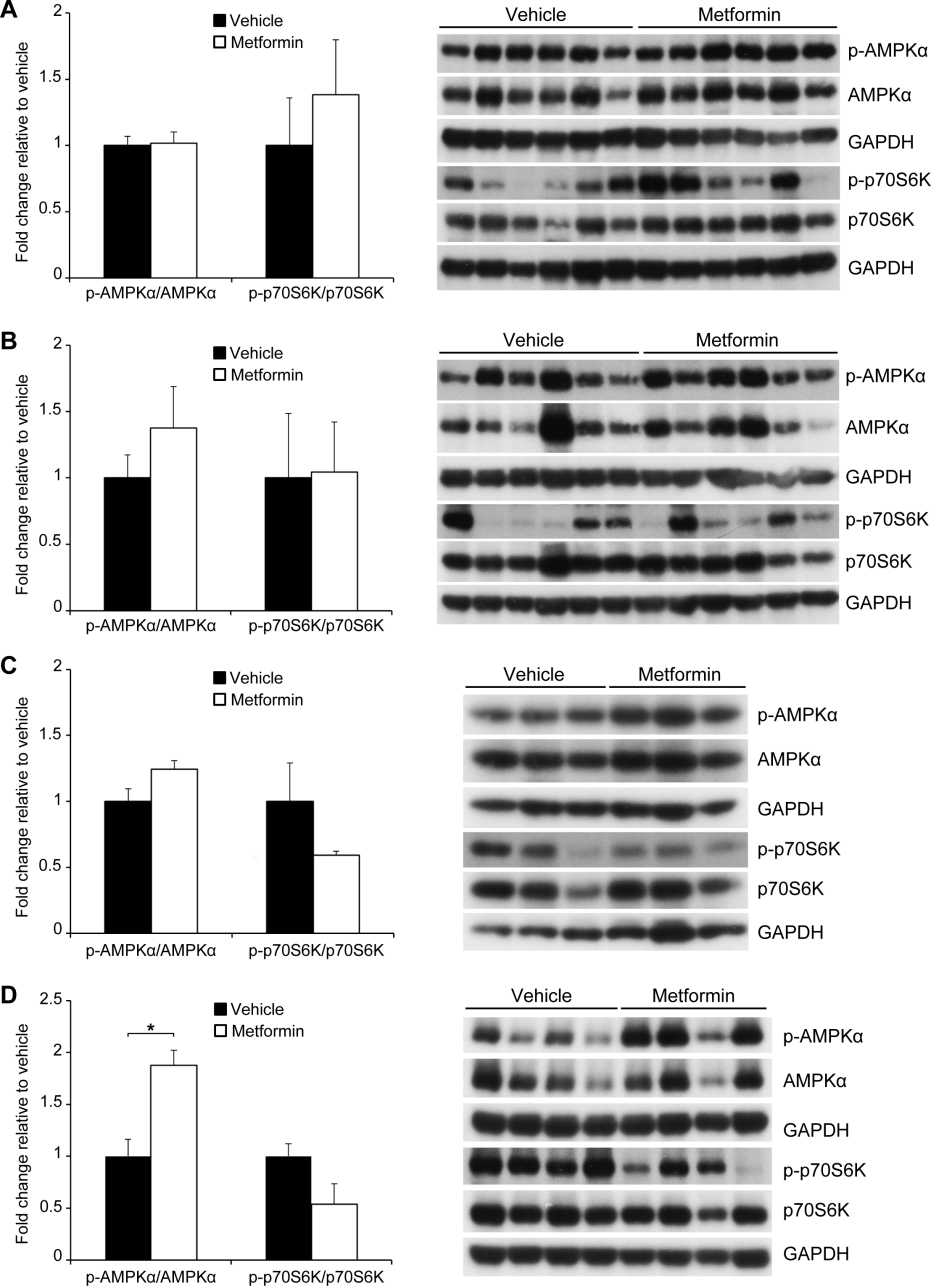
Activation of AMPK and inhibition of p70S6K phosphorylation in PDX tumors is not sustained after 28 days of metformin treatment. Phosphorylation of AMPKα and p70S6K in (A) P505 and (B) P710 PDX tumors after 28 day treatment with 400 mg/kg metformin. Phosphorylation of AMPKα and p70S6K in (C) P505 and (D) P710 PDX tumors after 3 day treatment with 400 mg/kg metformin (*p<0.05).

To determine whether metformin treatment altered AMPK and mTOR signaling, we evaluated all four PDX tumor lines for phosphorylation of AMPK (Thr172) and p70S6K (Thr389) at the end of the 28-day treatment (Fig 2A and 2B and S2 Fig). In this long-term treatment cohort, no change in phosphorylation of AMPK and p70S6K was seen in the metformin compared to the vehicle treated tumors. We then evaluated the effect of metformin on two PDX tumors after only 3 days of treatment. In contrast to the long-term treatment tumors, the short-term treatment tumors showed increased phosphorylation of AMPK and decreased phosphorylation of p70S6K (Fig 2C and 2D).

### Metformin inhibits growth and alters AMPK and mTOR signaling in pancreatic cancer cell lines

**Fig 3:**
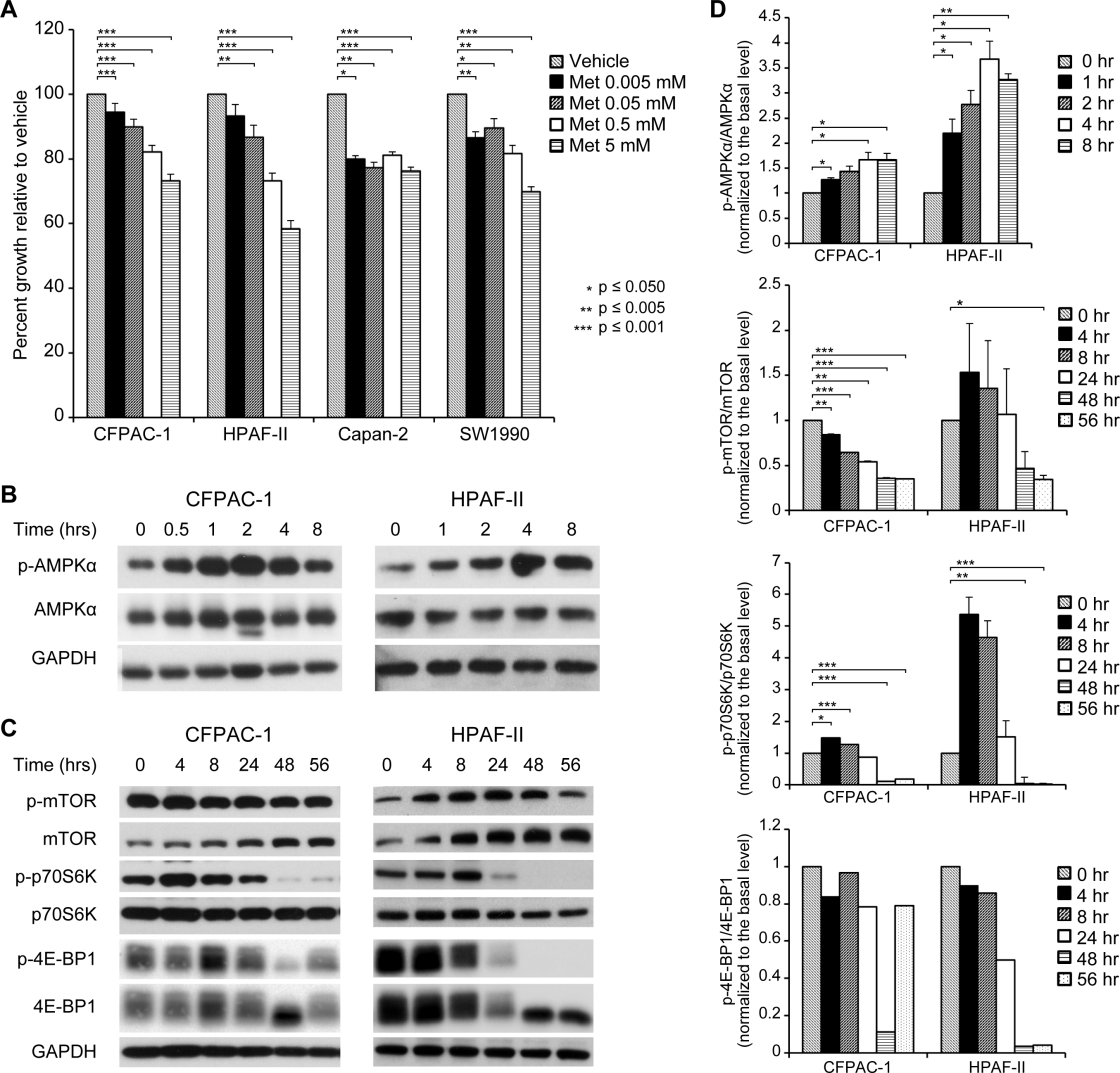
Metformin inhibits growth and alters AMPK and mTOR signaling in pancreatic cancer cell lines. (A) Cells were plated in quadruplicate into 96-well plates at a density of 5x10^3^ per well, incubated overnight, and then treated with media containing either PBS as a vehicle control or different concentrations of metformin (0–5 mM). After 48 hours, proliferation indices were determined using the MTT assay and normalized to those of the vehicle-treated cells. All assays were performed in triplicate. (B) Phosphorylation of AMPKα and total AMPKα at various time points after treatment with 5 mM metformin. Treatment began at 0 hours (hrs). (C) Phosphorylation of mTOR, p70S6K, and 4E-BP1 at various time points after treatment with 5 mM metformin. (D) Densitometry of phosphorylated AMPKα, mTOR, p70S6K, and 4E-BP1 relative to total levels shown in (B) and (C).

Since the lack of sustained response in our PDX models was surprising, we next examined the effects of metformin on the proliferation of four pancreatic cancer cell lines (Capan-2, CFPAC-1, HPAF-II, and SW1990). Metformin inhibited cell proliferation in a dose-dependent fashion in all four cell lines (Fig 3A). Metformin treatment activated AMPK as determined by phosphorylation of AMPK at Thr(172) in all cell lines tested, with a peak activation occurring at 4–8 hours after treatment (Fig 3B). Given that AMPK activation is known to inhibit mTOR, we further analyzed the effects of metformin treatment on the phosphorylation status of mTOR and its downstream targets p70S6K and 4E-BP1. Interestingly, we observed a delayed inhibition of mTOR and downstream target phosphorylation, with the nadir of observed phosphorylation of p70S6K and 4E-BP1 occurring at 48 hours in both CFPAC-1 and HPAF-II cells (Fig 3C and 3D).

### AMPK is only partially required for the anti-proliferative effect of metformin

**Fig 4:**
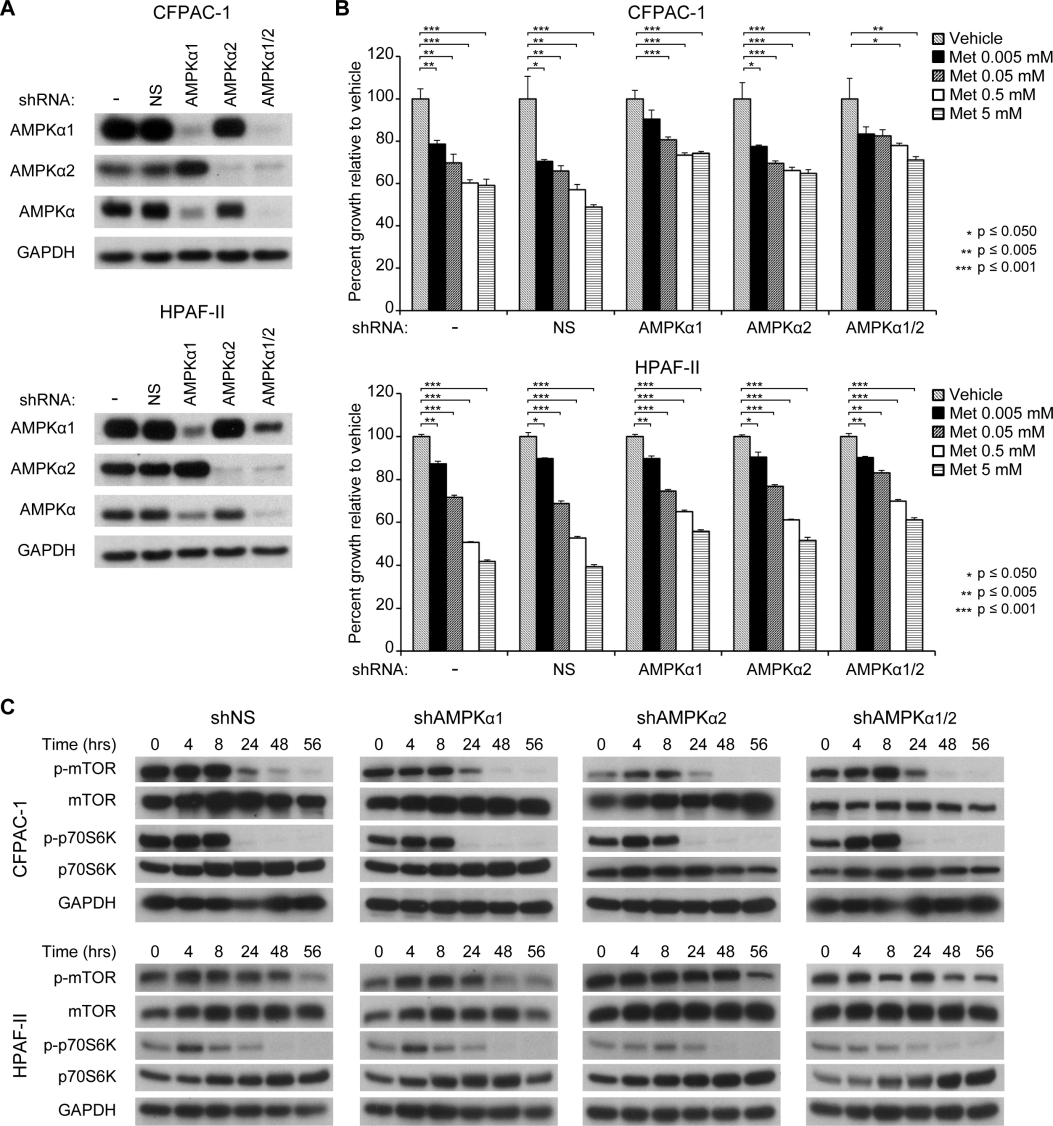
AMPK is only partially required for the anti-proliferative effects of metformin. (A) shRNA knockdown of AMPKα subunits in CFPAC-1 and HPAF-II cell lines. (B) Proliferation of CFPAC-1 and HPAF-II cell lines with stable knockdown of AMPKα subunits after treatment with different concentrations of metformin (0–5 mM). (C) Phosphorylation of mTOR and p70S6K in CFPAC-1 and HPAF-II cell lines with stable knockdown of AMPKα subunits.

The anti-proliferative effect of metformin has been primarily attributed to its ability to activate the AMPK pathway. We hypothesized that the lack of sustained AMPK activation seen in all four PDX tumor lines may explain the lack of tumor growth response. Thus, we performed shRNA-induced knockdown of one or both of the catalytic subunits of AMPK in pancreatic cancer cell lines to determine whether the anti-proliferative effects of metformin would be affected. We found that knockdown of AMPK α1 and/or AMPK α2 partially but not completely reversed metformin’s ability to inhibit cell proliferation (Fig 4A and 4B).

### Metformin appears to act on mTOR in an AMPK-independent manner

We next investigated whether the lack of sustained p70S6K inhibition seen in the P710 PDX tumor line despite evidence of some sustained AMPK activation may have been due to an AMPK-independent mechanism. We evaluated the effect of metformin on phosphorylation of mTOR and p70S6K following knockdown of AMPK α1 and/or AMPK α2. CFPAC-1 and HPAF-II cell lines with stable knockdown of NS, AMPK α1 and/or AMPK α2 (Fig 4A) were treated with 5 mM metformin (Fig 4B and 4C). In both cell lines, knockdown of one or both subunits did not rescue the ability of metformin to inhibit growth or phosphorylation of mTOR and p70S6K, suggesting that the ability of metformin to inhibit mTOR and p70S6K is at least partially independent of AMPK activation in these cell lines.

### mTOR overexpression is sufficient to overcome the anti-proliferative effects of metformin, but combinatorial treatment with metformin and mTOR inhibitors does not produce synergy

**Fig 5:**
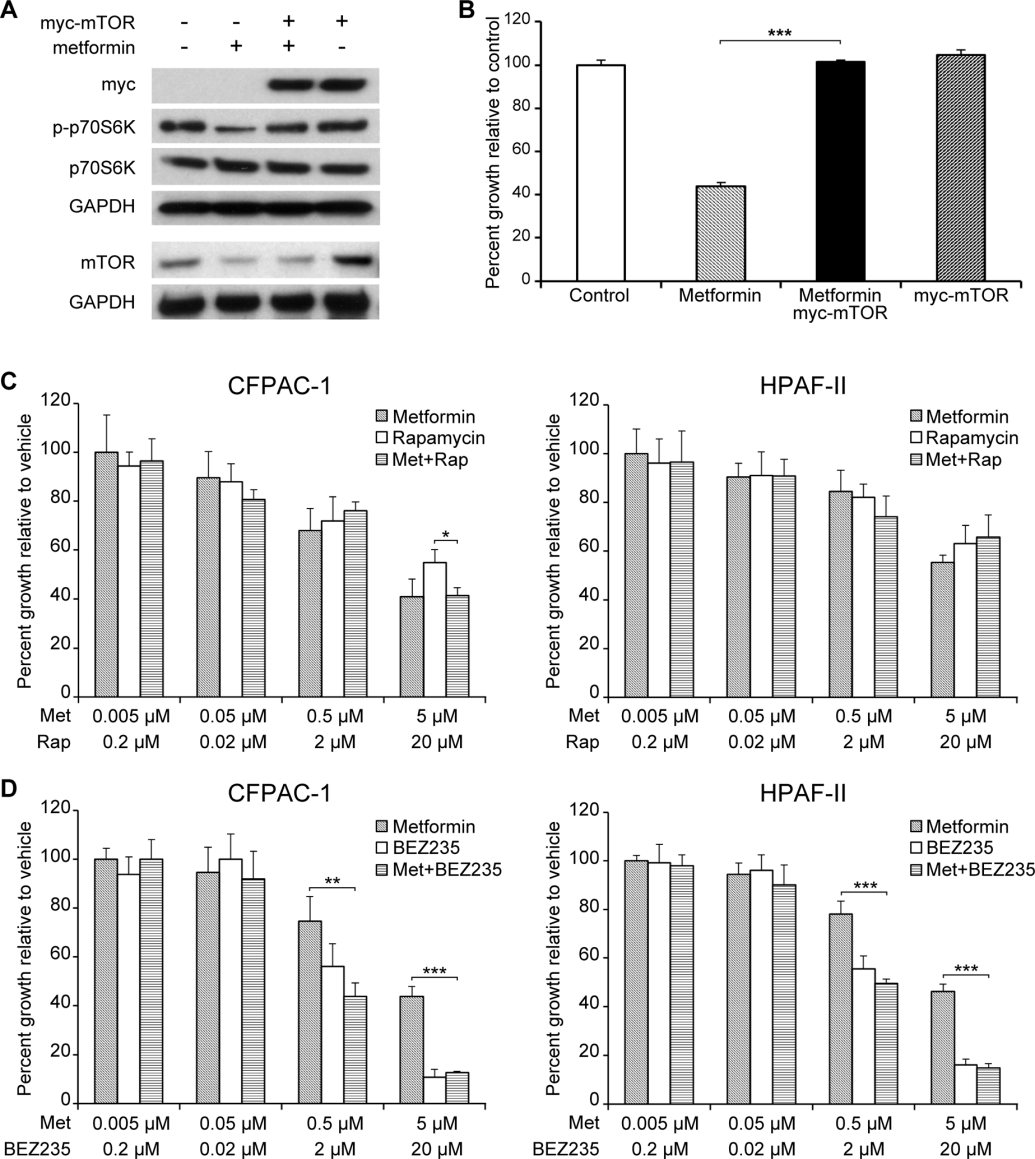
mTOR expression is sufficient to promote resistance to metformin in cell lines, but combinatorial treatment with metformin and mTOR inhibitors does not produce synergy. (A) Phosphorylation of p70S6K in and (B) proliferation of HPAF-II cells after treatment with 5 mM metformin following transient expression of a transfected myc-mTOR construct. Using the median effect equation calculation for combination index (CI) following 3 day treatment with constant-ratio doses of metformin and either (C) the allosteric mTOR inhibitor rapamycin or (D) the catalytic mTOR inhibitor BEZ235 failed to produce synergy (CI<1) at any dose combination. CI values at 50% growth inhibition: (C) CFPAC-1 1.54, HPAF-II 1.43; (D) CFPAC-1 1.30, HPAF-II 1.29. (*p<0.050, **p<0.005, ***p<0.001).

Because metformin failed to sustain inhibition of the mTOR pathway as measured by p70S6K phosphorylation in our long-term metformin treated PDX tumors, and because of our findings that metformin-induced mTOR inhibition was at least partially AMPK-independent, we next determined whether mTOR activation alone was sufficient to abrogate the effects of metformin. We found that overexpression of mTOR using a myc-mTOR construct was sufficient to produce complete resistance growth inhibition and to limit the decrease in p70S6K phosphorylation following metformin treatment (Fig 5A and 5B). To determine whether combining metformin with targeted mTOR inhibitors may be a logical therapeutic strategy, we treated cell lines with constant-ratio doses of metformin and either the allosteric mTOR inhibitor rapamycin or the catalytic mTOR inhibitor BEZ235, which are known to inhibit pancreatic cancer cell line growth. Growth inhibition was not enhanced at any dose combination relative to single drug treatment, and no synergism between metformin and either mTOR inhibitor was calculated at any dose using the Chou-Talalay median effect equation (Fig 5C and 5D).

## Discussion

Epidemiological studies in diabetic patients have found that patients treated with metformin have a decreased incidence of multiple cancers, including pancreatic cancer [14–17]. Several preclinical studies of metformin as an anti-cancer therapeutic have been promising, demonstrating impressive tumor growth inhibition [20, 22, 33] and apoptosis [34] of pancreatic cancer cell lines. However, cell line xenografts have been generally unreliable predictors of drug responses in humans. Lonardo et al. evaluated the effect of metformin on four pancreatic cancer PDX tumor lines and, similar to previous cell line xenograft studies, found substantial growth inhibition [21]. In contrast, emerging clinical trials evaluating metformin in pancreatic cancer have tempered the optimism created by this preclinical work. A double-blind, randomized, placebo-controlled phase II trial evaluating metformin in combination with gemcitabine and erlotinib in patients with advanced pancreatic cancer showed no difference in outcome as a result of metformin treatment [35]. Another phase II trial combining metformin with paclitaxel in patients with gemcitabine-refractory disease failed to meet its primary endpoint of disease control rate [36].

In this study, we observed a uniform lack of response to metformin in the four PDX tumor lines that we evaluated. Several potential reasons exist for the disparate results between previous preclinical work compared to our study and recent clinical trials. First, PDX tumors are inherently highly heterogeneous because PDX tumors are passaged in bulk and are representative ‘biopsies’ of similarly heterogeneous source patient tumors. Second, although the dose range in our study overlaps with previous studies, the pharmacokinetics of metformin uptake are still unclear [37]. Tumor microenvironment and stromal content appear to influence metformin’s access to tumor cells, which may lead to different outcomes between studies [21]. Third, the tumor volume at which treatment was initiated varies between studies, which may affect tumor composition, specifically the cancer stem cell burden. Lonardo et al. and others have shown that only this stem cell subpopulation undergoes apoptosis as a result of metformin treatment, while the vast majority of tumor cells experience reversible growth arrest [18, 21]. Interestingly, although Lonardo et al. found that metformin was able to initially slow PDX tumor growth, they noted that all PDX tumors eventually progressed on therapy [21], suggesting that metformin monotherapy will not be effective in patients.

To gain insight into possible mechanisms of resistance, we further evaluated two of our four PDX lines and found that resistance to metformin may be multifactorial and tumor-dependent. For example, in P505, AMPK activation was not sustained whereas in P710, continued tumor growth occurred despite some sustained AMPK activation. Our subsequent results in cell lines suggest that this may be for two reasons. First, the effect of metformin on cell proliferation appears to be only partially AMPK-dependent. Second, the ability of metformin to inhibit the mTOR pathway in pancreatic cancer also appears to be only partially AMPK-dependent. Across cancer types, the degree to which metformin relies upon AMPK activation to inhibit growth and alter mTOR/p70S6K signaling is unclear and likely cell type-dependent. Inhibition of AMPK expression via silencing of the catalytic AMPK α subunit using specific inhibitors of AMPK or knockout of LKB1, the upstream signal for AMPK activation, reversed the anti-proliferative effects of metformin in breast and ovarian cancer cells [38–40]. Conversely, Ben Sahra et al. showed that downregulation of AMPK had no effect on metformin’s ability to inhibit prostate cancer cell growth and mTOR signaling [41]. Instead, they found that metformin led to mTOR inhibition and cell-cycle arrest by activating the mTORC1 inhibitor REDD1 [42]. Other studies in breast and ovarian cancer cell lines have also found that the effects of metformin may be only partially dependent on AMPK activation [43, 44]. The anti-proliferative effect of metformin was maintained in the ovarian cancer cell line A2780 despite siRNA silencing of AMPKα1. In addition, metformin treatment was able to attenuate proliferation of both AMPKα1/2 wild-type and AMPKα1/2 deficient mouse embryonic fibroblasts (MEFs), although AMPKα1/2 deficient MEFs were slightly less sensitive to metformin [43]. In breast cancer cell lines, inhibition of HER2 by metformin was found to be completely AMPK-independent [44]. Recent studies in pancreatic cancer cell lines found that metformin may inhibit growth independently of AMPK through upregulation of miR-26a [45], while metformin’s effects on pancreatic cancer stem cells may be mediated through reexpression of specific miRNAs [18]. These results suggest that modulation of miRNA expression may be yet another important mechanism underlying the biological effects of metformin.

Taken together with the above studies, our results that knockdown of AMPK subunits did not rescue the inhibitory effects of metformin on mTOR/p70S6K phosphorylation but that mTOR reexpression was able to reverse the anti-proliferative effects of metformin, suggest that the activation of AMPK and inhibition of the mTOR/p70S6K pathway by metformin are independent events that may both contribute to cancer cell growth inhibition. Moreover, regulation of AMPK by metformin may be cell type dependent, and in pancreatic cancer, the anti-proliferative effects of metformin may be partially or largely AMPK-independent.

Overall, our study shows that although metformin inhibits pancreatic cancer cell line proliferation, its effect on patient tumors will likely be transient and much more complex. While resistance mechanisms likely involve mTOR pathway activation, simultaneous treatment with metformin and mTOR inhibitors may do little to enhance the efficacy of either therapy. Further studies are needed in order to determine whether metformin may someday provide benefit to pancreatic cancer patients by leveraging its complex metabolic and signaling effects in combination with chemotherapeutics or targeted therapies.

## Supporting Information

S1 Fig Long-term metformin treatment does not affect mouse weight or tumor histology. (A) Weights of mice over a 28 day treatment course with either 200 mg/kg or 400 mg/kg metformin shown relative to baseline weights. Hematoxylin and eosin staining of (B) P505 and (C) P710 patient-derived xenograft tumors following 28 day treatment with vehicle (left panels) or 400 mg/kg metformin (right panels) show no difference in tumor architecture, ductal formation, or stromal content. Scale bars are 300 μm.
(TIF)

S2 Fig Activation of AMPK and inhibition of p70S6K phosphorylation in PDX tumors is not sustained after 28 days of metformin treatment. Phosphorylation of AMPKα and p70S6K in (A) P722 and (B) PT4 PDX tumors after 28 day treatment with 400 mg/kg metformin.
(TIF)
