# Supplementary figures and images for: Metformin Treatment Does Not Inhibit Growth of Pancreatic Cancer Patient-Derived Xenografts
Source: PLoS One. 2016 Jan 13;11(1):e0147113. doi: 10.1371/journal.pone.0147113 (PMC4711922; doi:10.1371/journal.pone.0147113)

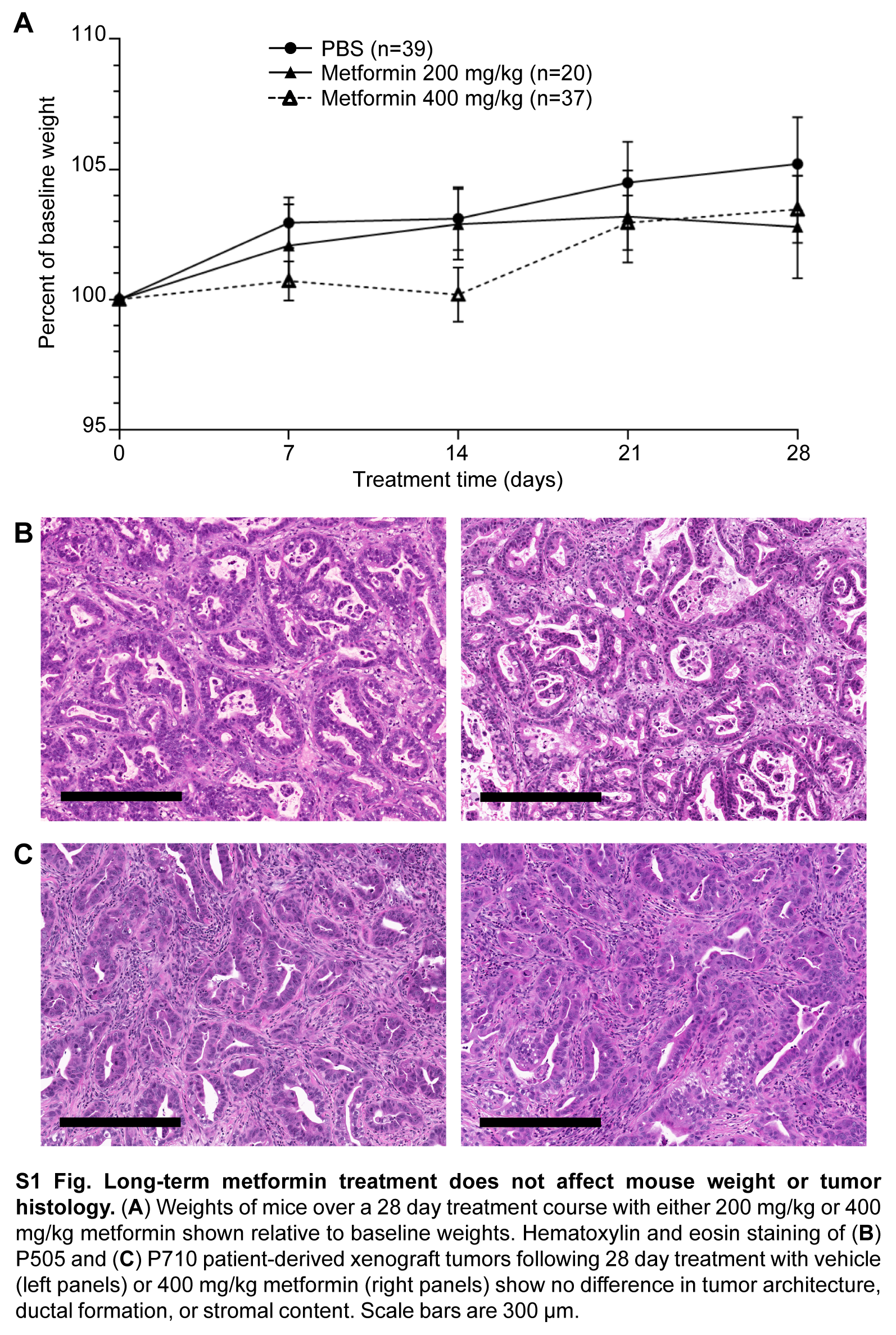

Supplement: S1 Fig — (A) Weights of mice over a 28 day treatment course with either 200 mg/kg or 400 mg/kg metformin shown relative to baseline weights. Hematoxylin and eosin staining of (B) P505 and (C) P710 patient-derived xenograft tumors following 28 day treatment with vehicle (left panels) or 400 mg/kg metformin (right panels) show no difference in tumor architecture, ductal formation, or stromal content. Scale bars are 300 μm. (TIF) [file pone.0147113.s001.tif]

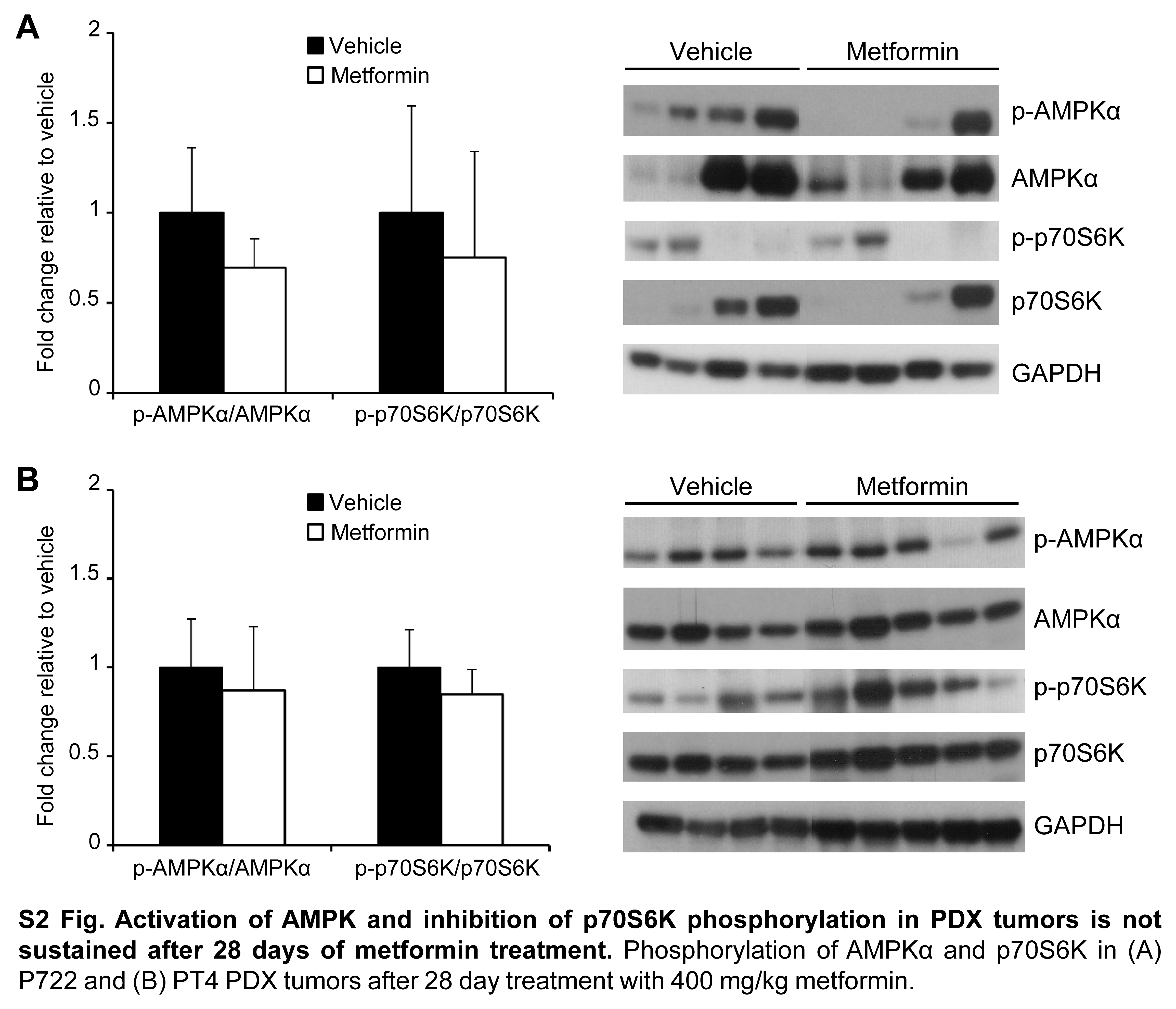

Supplement: S2 Fig — Phosphorylation of AMPKα and p70S6K in (A) P722 and (B) PT4 PDX tumors after 28 day treatment with 400 mg/kg metformin. (TIF) [file pone.0147113.s002.tif]
